# Cytotoxic Activities and Fingerprint Analysis of Triterpenes by HPTLC Technique for Distinguishing *Ganoderma* Species from Vietnam and other Asian Countries

**DOI:** 10.3390/plants11233397

**Published:** 2022-12-06

**Authors:** Tran Viet Hung, Phan Nguyen Truong Thang, Ha Minh Hien, Vu Thi Diep, Nguyen Thi Thu, Duong Minh Tan, Duy Toan Pham, Do Thi Ha, Duyen Thi My Huynh

**Affiliations:** 1 Institute of Drug Quality Control-Ho Chi Minh City (IDQC HCMC), Ho Chi Minh City 700000, Vietnam; 2 National Institute of Medicinal Materials (NIMM), Hanoi 100000, Vietnam; 3 National Institute of Drug Quality Control (NIDQC), Hanoi 100000, Vietnam; 4 Department of Chemistry, College of Natural Sciences, Can Tho University, Can Tho 900000, Vietnam; 5 Department of Pharmaceutical and Pharmaceutical Technology, Faculty of Pharmacy, Can Tho University of Medicine and Pharmacy, Can Tho 900000, Vietnam

**Keywords:** *Ganoderma* species, HPTLC fingerprint, cytotoxicity, triterpenoids

## Abstract

*Ganoderma lucidum* (Fr.) P. Karst. (Ganodermataceae), commonly called Linhzhi, is traditionally employed in the treatment of human diseases, including hepatitis, liver disorders, hypercholesterolemia, arthritis, bronchitis, and tumorigenic diseases. In this study, the fingerprint profiles of five different strains of *G. lucidum* originated from Japan, Korea, China, and Vietnam, five samples of *G. lucidum* growing on *Erythrophloeum fordii* Oliv. in Vietnam, and five related Linhzhi species (*Ganoderma applanatum*, *Ganoderma australe*, *Ganoderma clossum*, *Ganoderma subresinosu*, and *Ganoderma sp.*) were investigated for triterpene derivatives using high-pressure, thin-layer chromatography (HPTLC). The HPTLC fingerprint profiles demonstrated significant differences between *G. lucidum* and other related Linhzhi species in the presence of triterpene derivatives. Evaluation for the cytotoxicity of these samples against four cancer cell lines, including A549, MCF7, PC3, and HepG2, displayed various levels of cytotoxic effects, with IC_50_ values of: 15.6–46.3 µg/mL on the A549 cancer cell line, of 18.4–43.6 µg/mL on the MCF7 cancer cell line, of 10.0–32.1 µg/mL on the PC3 cancer cell line, and of 10.6–27.6 µg/mL on the HepG2 cancer cell line. Conclusively, these data contributed to the literature on the cytotoxic activities and fingerprint analysis of triterpenes by the HPTLC technique for distinguishing *Ganoderma* species from Vietnam and other Asian countries.

## 1. Introduction

Botanical studies have considerably increased in recent years, especially in the plant and mushroom areas [1,2]. Among various investigated mushrooms, *Ganoderma lucidum* (Leyss. Ex Fr.) P. Karst (*G. lucidum,* Linhzhi) is a traditional herb that is commonly and ethnopharmacologically utilized in Asian countries for its outstanding beneficial activities in many diseases, such as insomnia, dizziness, anorexia, hypercholesterolemia, chronic hepatitis, coronary heart disease, carcinoma, and hypertension [3,4,5,6,7,8]. Linhzhi was deemed as a tincture of life for thousands of years, and nowadays, a number of commercial Linhzhi products such as tea, capsules, tablets, raw herb, powder, and extract are found in the market places as remedies for the treatment of different diseases, such as coronary heart diseases, arteriosclerosis, hepatitis, arthritis, nephritis, bronchitis, asthma, hypertension, cancer, and gastric ulcer [3,4]. Modern uses of *Garnoderma* include treatment of immunomodulatory effect [9], antitumor activity [9,10], anti-inflammation [11], cardiovascular activity [12], liver protection and detoxification [13,14], hepatitis, and gastric ulcer [3,4,13,14]. Modern research has clarified a variety of chemical ingredients, including polysaccharides, triterpenes, nucleotides, sterols, steroids, fatty acids, proteins, and peptides [15]. Among them, the most important pharmacologically active constituents of *G. lucidum* are triterpenes and polysaccharides [3,4]. Triterpenes have received considerable attention owing to their well-known pharmacological activities, such as hepatoprotective, anti-hypertensive, anti-histaminic, antitumor, and anti-engiogenic activity [3,8,16,17,18,19]. In addition, ergosterol (provitamin D2) has been reported in concentrations of 0.3–0.4% in Reishi and had effects on H/R-induced oxidative stress and inflammatory response. Hence, triterpenes and ergosterol could be considered as the “marker compounds” for chemical evaluations or standardizations of *G. lucidum* [20,21].

Nevertheless, despite the valuable dietary and therapeutic benefits of Linhzhi species, chemical investigations of the active components have been conducted mostly in China, Republic of Korea, Japan, and the United States [5]. However, a few experiments demonstrating the medical properties of the local *Ganoderma* genus (the family: Ganodermataceae) have been carried out recently in Europe, India, and Iran [22,23,24,25]. So far, several studies have been reported on taxonomy and distribution properties of Ganodermataceae in Vietnam [26,27,28]. According to Dam et al., 1997, Vietnam possesses 26 species and 1 variety of *Ganodema* genus. In a recent study, 43 species of *Ganoderma* genus have been detected in the highlands of Vietnam. Six of these *Ganoderma* species have been successfully cultivated in Vietnam, including *G. lucidum*, *G. applanatum*, *G. australe*, *G. clossum*, *G. subresinosum*, and *G. sp*.

*Ganoderma* species are wood-decaying fungi that parasitize on dead or dried trunks of trees, mostly found in temperate forests. Recently, Vietnamese people have been using “Nấm Lim Xanh”, the *G. lucidum* that grows on *Erythrophloeum fordii* Oliv. from Quang Nam (Tien Phuoc, Tra My, and Dong Giang districts) for the treatment of liver cancer, which possesses a price around 3–4 times higher than cultivated Vietnamese Linhzhi species originated from Japan, Korea, and China in Vietnamese markets. However, there is no scientific study reported on their chemical compositions from the wild Vietnamese *G. lucidum* species and no comparison data to those of cultivated and other related Linhzhi species. Therefore, this is urgent work for scientists to evaluate cytotoxic activities against several cancer cell lines of cultivated Linhzhi originated from Japan, China, Korea, and Vietnam and five related Linhzhi species, including *G. applanatum*, *G. australe*, *G. clossum*, *G. subresinosum*, and *G. sp,* and the wild collected “Nấm Lim xanh”.

Nowadays, the profiling of the relative amounts of various active compositions (fingerprint profile) has been proven to be a convenient and effective method for qualitative and quantitative analysis and standardization of medicinal materials. Although HPLC is a popular method for fingerprint analysis of herbal medicines [29], the unique feature of picture-like images of HPTLC coupled with the digital scanning profile is becoming more attractive for herbal analysis to build up the herbal chromatographic fingerprint by means of HPTLC [30]. An important property of HPTLC fingerprint analysis is the large number of samples that can be analyzed in parallel [31]. Additionally, it could be used to establish proper extraction parameters, to standardize extracts, and to detect any changes or degradation in the material during formulation [32]. Therefore, this study using the fluorescence HPTLC fingerprint of triterpenes and an ergosterol in cultivated Linhzhi samples, wild-collected Linhzhi (Nấm Lim Xanh), and five related Linhzhi samples was developed, and the corresponding digital scanning chromatograms were created with self-developed software. Six lanostane triterpenes and an ergosterol isolated from wild-collected Linhzhi in Quangnam Danang were used as chemical reference substances. In addition, in vitro cytotoxic activities against four cancer cell lines (A549, MCF7, PC3, and HepG2) have also been examined.

## 2. Results

### 2.1. Selection of Extraction Method

Several methods for extraction of fruiting bodies of *Ganoderma* species were surveyed, such as sonication, reflux, and maceration (data not shown), using methanol and ethanol solvents. Among those, the extract with the highest yield was obtained using a reflux extraction method with a methanol solvent. Therefore, this method was used to prepare sample solutions for HPTLC fingerprint analysis and sample extracts for cytotoxic evaluation. Percentages of extract yields are demonstrated in Table 1.

### 2.2. HPTLC Analysis

**Figure 1:**
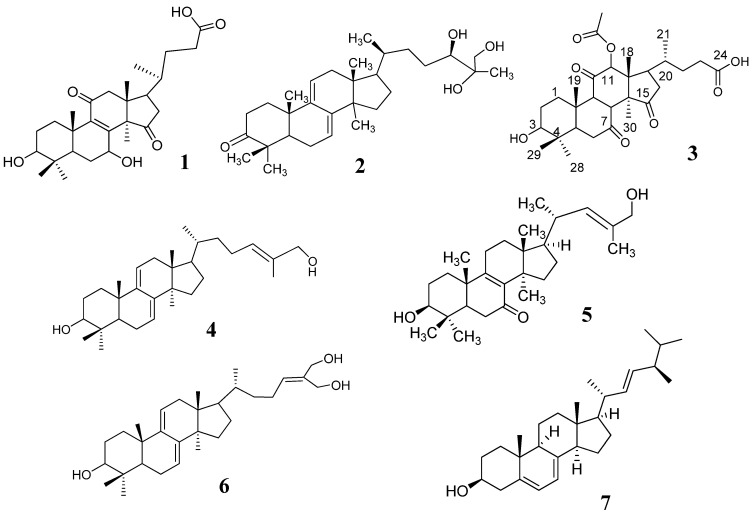
Chemical constituents isolated from wild-collected Linhzhi in Vietnam.

**Figure 2:**
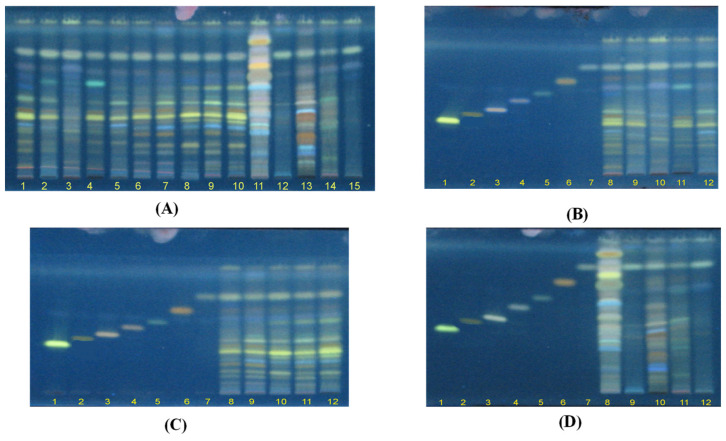
HPTLC fluorescence images under the excitation wavelength of 366 nm. (**A**) 15 samples of *Ganoderma* species (G1–G15). (**B**) G1–G5 and 7 chemical reference substances (CRS): lucidenic acid N (**1**), ganodermanontriol (**2**), lucidenic acid E2 (**3**), ganoderiol F (**4**), lucidadiol (**5**), ganodermadiol (**6**), and ergosterol (**7**). (**C**) G6–G10 and 7 CRS. (**D**) G11–G15 and 7 CRS.

Triterpenes and ergosterol (**1–7**, Figure 1) are nonpolar compounds; therefore, the solvent system that obtained the optimized resolution of the HPTLC images is dichloromethane:methanol (9:1). A high resolution with fluorescence bands in the chromatogram of 15 tested samples (G1–G15) was observed (Figure 2A). The chromatogram of samples G1–G10 showed 12–13 fluorescence bands with R_f_ values higher than 0.4, which were regarded as triterpenes and ergosterol derivatives. In comparison, chromatograms of samples G11–G15 were significantly different from each other and from the chromatograms of samples G1–G10.

Figure 2B–D present the chromatograms of G1–G5 and chemical reference substances (CRS), G6–G10 and 7 CRS, and G11–G15 and 7 CRS. The results showed that 7 CRS were presented in *G. lucidum* originated from Japan (G1), China (G2), Korea (G4), and Vietnam (G3 and G5), and wildly collected samples in Quang Nam, Vietnam (G6–G10). However, G5–G6 possessed a higher level of lucidenic acid N (**1**) and lucidadiol (**5**) compared to those in G1–G5. It is important to note that fluorescence bands with R_f_ values of 0.26–0.3 of G. *applanatum* (G11), *G. clossum* (G12), *G. subresinosum* (G13), *G. sp* (G14), and *G. australe* (G15) were different to those of *G. lucidum* (G1–G10) (Figure 2B–D). As demonstrated in Figure 2D, only ganodermadiol (**6**) and ergosterol (**7**) appeared in five *Ganoderma* species (G11–G15).

### 2.3. Fingerprint Profile of Different Ganoderma Species Extracts

**Figure 3:**
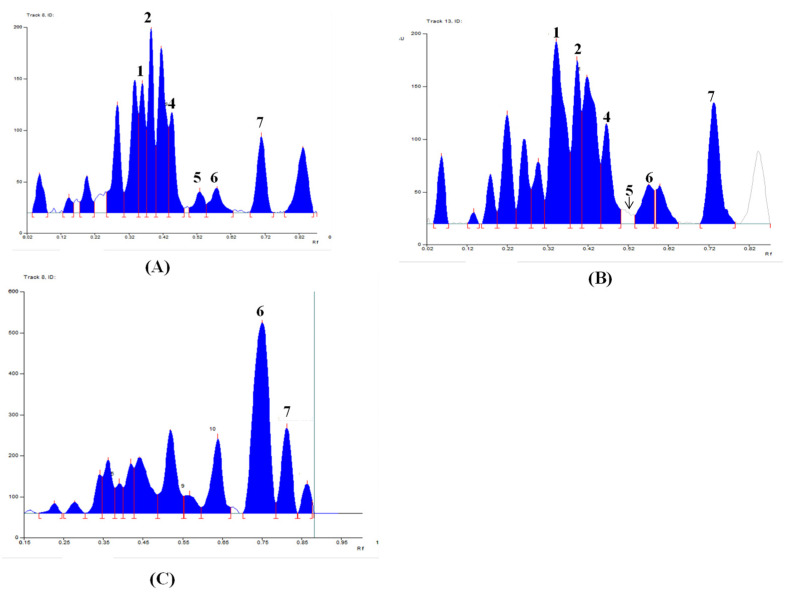
Typical HPTLC images and corresponding digital profiles of (**A**) G1–G5 and 7 chemical reference substances (CRS) of lucidenic acid N (**1**), ganodermanontriol (**2**), lucidenic acid E2 (**3**), ganoderiol F (**4**), lucidadiol (**5**), ganodermadiol (**6**), and ergosterol (**7**), (**B**) G6–G10 and 7 CRS, (**C**) G11–G15 and 7 CRS.

**Figure 4:**
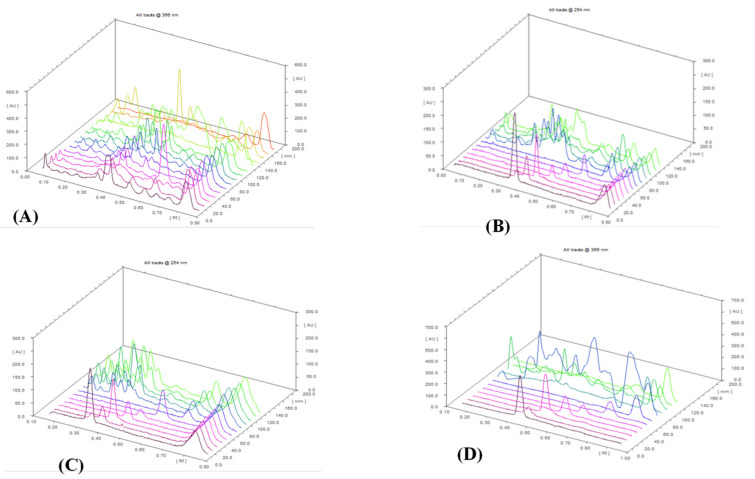
The quantifiable comparison of 3D graphs of the HPTLC fingerprint of 15 *Ganoderma* species and CRS. (**A**) 15 samples of *Ganoderma* species (G1–G15). (**B**) G1–G5 and 7 CRS: lucidenic acid N (**1**), ganodermanontriol (**2**), lucidenic acid E2 (**3**), ganoderiol F (**4**), lucidadiol (**5**), ganodermadiol (**6**), and ergosterol (**7**). (**C**) G6–G10 and 7 CRS. (**D**) G11–G15 and 7 CRS.

Among the 5 other Linhzhi species (G11–G15), only G11 (*G. applanatum*) presented the most fluorescence bands. Therefore, G11 and G1 (*G. lucidum* originated from Japan), and G6 (wild *G. lucidum* collected in Quang Nam, Vietnam), were evaluated for the fingerprint profiles. As shown in Figure 3, chromatograms of G1 (Figure 3A) were similar to those of G6 (Figure 3B). Seven triterpenes, including lucidenic acid N (**1**), ganodermanontriol (**2**), lucidenic acid E2 (**3**), ganoderiol F (**4**), lucidadiol (**5**), ganodermadiol (**6**), and ergosterol (**7**), appeared in both samples (G1 and G6, Figure 3A,B). However, only two compounds (**6** and **7**) appeared in G11 (Figure 3C). The quantifiable comparison of 3D graphs of the HPTLC fingerprint of Linhzhi (G1–G10) and the 5 related Linhzhi species (G11–G15) with 7 standard reference compounds (**1**–**7**) is depicted in Figure 4A–D. It is clear that the fingerprint profile of *G. lucidum* (cultivated and wildly collected) was very different than the other related Linhzhi species (G11–G15). It is also noted that the wildly grown Linhzhi species (G6–G10) yielded a fingerprint profile that contained a larger number of peaks and a different peak intensity profile when compared to those of cultivated Linhzhi samples (G1–G5), and with the other 5 related Linhzhi species (G11–G15). However, all analyzed samples contained ergosterol, a specific component of the fungal cell membrane.

### 2.4. In Vitro Cytotoxic Activity

Three cultivated Linhzhi (G1–G4), a wild-collected Linhzhi (G6), and five related Linhzhi species (G11–G15) have been evaluated for the inhibition of in vitro cytotoxic effects on four cancer cell lines, including A549, MCF7, PC3, and HepG2. Results are shown in Table 2.

According to the US NCI rules on the plant extracts/pure compounds’ in vitro cytotoxicity, a plant extract is considered to be toxic to a cell line if its IC_50_ value is <20 µg/mL after an incubation time of 48 h, whereas this value should be <10 µg/mL for the pure compounds [33]. As shown in Table 2, among the four cultivated Linhzhi, G1, G2, G3, and G4, which originated from Japan, China, Vietnam, and Korea, G1 showed a potent cytotoxic effect on PC3 and HepG2 cancer cell lines. G2 and G4 showed significant inhibitory activity on a PC3 cancer cell line and a moderate inhibitory activity on HepG2 and A549 cancer cell lines. G1, G2, and G4 displayed non-cytotoxicity against the MCF7 cancer cell line, with an IC_50_ value > 50 µg/mL. G3 presented a moderate inhibitory activity on the MCF7 cell line, with an IC_50_ value of 33.8 ± 3.4 µg/mL, and was inactive on A549 and PC3, with IC_50_ values > 50 µg/mL. For the comparison between cultivated Linhzhi (G1–G4) and a Linhzhi sample collected from nature (G6), a similar effect on the inhibition of three cancer cell lines (MCF7, PC3, and HepG2) was observed. A difference was seen in the highest inhibitory activity of G6 on the A549 cell line, with an IC_50_ value of 9.12 ± 1.5 µg/mL, as compared to those of G1–G4.

Five related Linhzhi species, including *G. applanatum* (G11) and *G. clossum* (G12), displayed moderate cytotoxic activity against the A549 cancer cell line with IC_50_ values of 46.3 and 24.8 µg/mL, respectively. *G. subresinosum* (G13) and *G. sp* (G14) showed considerable cytotoxic activities against the A549 cancer cell line with IC_50_ values of 15.6 and 17.7 µg/mL. *G. australe* (G15) exhibited cytotoxic inactivity on this cell line at IC_50_ > 50 µg/mL. G13–G15 displayed significant cytotoxic activity against the MCF7 cancer cell line with IC_50_ values within 18.4–30.7 µg/mL. G11–G12 did not demonstrate any significant cytotoxic activity against the MCF7 cancer cell line. Four samples (G12, G13, G14, and G15) displayed a moderate inhibitory effect on PC3 with IC_50_ values ranging from 23.6 to 32.1 µg/mL, and G11 presented no cytotoxic activity on this cell line. G11, G13, and G15 did not show any significant cytotoxic activity against the HepG2 cell line. On the other hand, G12 and G14 displayed considerable cytotoxic effects against HepG2, with IC_50_ values of 20.2 and 23.5 µg/mL, respectively.

## 3. Discussion

In the present work, we have reported, for the first time, the fingerprint profiles of four Linhzhi strains originated from Vietnam, Japan, and China that were successively cultivated in Vietnam, one Korean Linhzhi strain that was cultivated in Korea, five wild-harvesting Linhzhi in Vietnam, and five related Linhzhi strains that were successively cultivated in Vietnam. It can be implied that different strains possess different chemical constituents due to differences in the geographical distributions, growth conditions, and substrates. In our study, the profiles of lanostan triterpenes differed considerably in the five different strains (G1–G10) and were distinguishable from the five related Linhzhi species (G11–G15). Similar data have been reported in the literature. The growth conditions might be the major factors contributing to the differences between the Iranian and Chinese Linhzhi strains in producing various ganoderic acids [24]. Besides, the quality assessment of Linhzhi and its respective commercial products was performed based on small (triterpenes and nucleic acids) and macro (polysaccharides) molecular bioactive compounds by using HPLC, high-performance size-exclusion chromatography evaporative light scattering detector (HPSEC-ELSD), and HPTLC. The data also displayed the obvious variations among Linhzhi strains or products [34,35]. Notably, the Italian *G. lucidum* possesses different phytochemical contents, namely protein and polysaccharide, compared to the same Chinese species cultivated in similar medium. Thus, this fact re-confirms that the bioactive components and the therapeutic activities of Linhzhi are heavily dependent on the climatic and geographical conditions. In our research, phytochemical investigation demonstrated significant differences between Linhzhi strains and related Linhzhi species. In addition, the dissimilarity of cytotoxicity against four human cancer cell lines: A549, MCF7, PC3, and HepG2, was observed from ten Linhzhi strains and five related Linhzhi species cultivated in Vietnam.

## 4. Materials and Methods

### 4.1. Plant Materials

**Figure 5:**
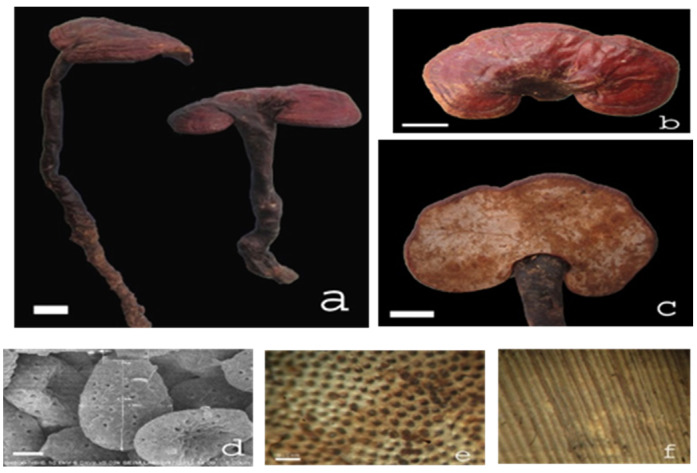
*Ganoderma* sample (G6, *Ganoderma lucidum* (Curtis) P. Karst. 1881), known locally as Natural Green Lim mushroom (Nấm Lim Xanh). Notes: (**a**–**c**) Fruit body, (**d**) spores (SEM), (**e**) planned layer, and (**f**) mushroom tube. Natural shape ruler = 2 cm, microscopic ruler = 2 μm, laminar ruler = 0.5 mm.

*Ganoderma* species samples (15 samples): The fruiting bodies of *Ganoderma lucidum* (G1–G3), *Ganoderma applanatum* (G11), *Ganoderma clossum* (G12), *Ganoderma subresinosum* (G13), *Ganoderma sp.* (G14), and *Ganoderma australe* (G15) were gifts from Linh chi Vina Company, Vietnam. *Ganoderma lucidum* (G4) was purchased from Longevity Linhzhi Farm, Korea. *Ganoderma lucidum* (G5) was purchased from the Vietnam Academy of Agricultural Sciences. *Ganoderma lucidum* (G6–G10) was wildly collected from Tienphuoc district (or Tien Phuoc), Quangnam province (or Quang Nam), Vietnam, known locally as Natural Green Lim mushroom (Nấm Lim Xanh). The samples (G1–G3, G11–G15) were botanically identified (Table 1) by Msc. Co Duc Trong, Linh chi Vina Company, Vietnam, where the voucher specimens were deposited. Specimens of the test samples (G6–G10) have been verified by Prof. Bae KiHwan from the College of Pharmacy, Chungnam National University, and deposited in the Department of Phytochemistry, National Institute of Medicinal Materials (NIMM), Vietnam. Sample G6 was collected growing on *Erythrophloeum fordii* Oliv. from Quang Nam (Tien Phuoc), and restored in both NIMM and the Institute of Drug Quality Control, Ho Chi Minh City (IDQC HCMC). All dried samples were ground and transferred to the laboratory for preparation of the plant extracts. The morphological and microscopic images of sample G6 are shown in Figure 5.

### 4.2. Cell Lines, Chemicals, and Reagents

For the cell culture chemicals, the RPMI-1640 medium, Dulbecco’s modified Eagle’s medium (DMEM), fetal bovine serum (FBS), and trypsin were imported from GIBCO-BRL (Grand Island, NY, USA). The chemical for the cytotoxicity assay, 3-(4,5-Dimethylthiazol-2-yl)-2,5-diphenyltetrazolium bromide, a tetrazole) (MTT), was bought from Sigma (St Louis, MO, USA). The MCF-7 (breast cancer), A549 (lung cancer), and PC3 (prostate cancer) cell lines were imported from RIKEN Cell Bank (Ibaragi, Japan). The HepG2 (hepatocellular carcinoma) cell line was purchased from the American Type Culture Collection (ATCC, Manassas, Virginia, USA). The A549 and PC3 cells were grown in RPMI containing 10% FBS. The HepG2 and MCF-7 cells were grown in DMEM containing 10% FBS. These cell lines were cultured at 37 °C in a humidified CO_2_ incubator [36]. All other chemicals and solvents used were of analytical grade or higher.

### 4.3. Instrumentations

Analytical glassware (pipets, beakers, conical flasks, volumetric flasks, etc.) were calibrated, refluxed, washed in a water bath, and used on a thermostatic stove or in a refrigerator. Chromatography glass columns: silica gel 60 for column chromatography (70–230, 230–400 mesh, Merck), and YMC gel for LC (ODS-A, 12 nm, 75 µm, AA12S75). High-performance, thin-layer chromatography (HPTLC) was conducted in the Camag TLC system (Camag, Muttenz, Switzerland) supplied with WinCATS software (version 1.2.3). The samples were run using an automatic TLC sampler 4 (STS 4), on HPTLC precoated silica gel plates (20 × 10 cm, Merck, Germany) in a glass chamber (24.5 × 8 × 22.5 cm). The TLC chromatogram imaging and documentation were recorded by a ReproStar 3 with VideoStore 2 software, Bruker NMR 500 Mhz, Institute of Chemistry, Vietnam Academic of Science and Technologies (VAST).

### 4.4. Extract Preparations

#### 4.4.1. Preparation of Extracts for Cytotoxic Test

The air-dried fruiting bodies of each sample of *Ganoderma* species (50 g) (summary in Table 1) were extracted with MeOH (3 × 0.6 L). Each extract was passed through a No. 1 Whatman filter (Whatman Inc., Hillsboro, OR, USA) and the filtrate was evaporated to dryness under a vacuum at 40 °C to obtain the MeOH extracts. The sample extracts were stored at −20 °C until the cytotoxic test.

#### 4.4.2. Preparation of Sample Solution for HPTLC Analysis

For the HPTLC analysis, 0.5 g of powder of each *Ganoderma* species was accurately weighted into a conical flask, respectively, and refluxed with 60 mL of MeOH for 1 h. The extract solution was filtered, and the residues were washed with 20 mL of MeOH, twice. This extraction process was repeated two times. The extracts were then combined and concentrated to dryness under a vacuum. The dried residue was dissolved in 2 mL of MeOH, filtered through a 0.45 µm membrane filter, and subjected to HPTLC analysis.

#### 4.4.3. Extraction and Isolation Marker Compounds

Seven kg of *G. lucidum* (G6) was extracted with MeOH (3 × 30 L) over 2 h, filtered, and concentrated to yield the MeOH extract (448 g). This extract was dispersed in water (1.5 L) and extracted with *n*-hexane (2 × 1.5 L) and CH_2_Cl_2_ (3 × 2.0 L). Then, the obtained CH_2_Cl_2_ extract (220 g) was dissolved in MeOH and filtered to obtain a precipitate, which was crystalized in CH_2_Cl_2_ to obtain compound **7** (1200 mg). The remaining filtrate (GLC2) was fractionated using silica gel column chromatography (60 × 12 cm; CH_2_Cl_2_–MeOH 200:1→1:1) to yield ten fractions (GLC2.1–GLC2.10). The fraction GLC2.3 (3.8 g) was purified by silica gel column chromatography (30 × 12 cm; *n*-hexane:EtOAc 20:1→1:1) to obtain compound **2** (399 mg). The fraction GLC2.4 (63.6 g) was chromatographed on a silica gel column (80 × 6 cm) and eluted with *n*-hexane–MeOH (10:1→2:1) to yield compound **6** (50 mg). The fraction GLC2.6 (63 g) was separated by silica gel column chromatography (CH_2_Cl_3_−MeOH, 20:1) to yield two sub-fractions, GLC2.6.1 and GLC2.6.2. The sub-fraction GLC2.6.2 (2.1 g) was further eluted through a YMC column (MeOH−H_2_O, 3:1) to generate compound **4** (10 mg). Fraction GLC2.7 (32 g) was separated with silica gel column chromatography using increasing polarity solvents (CH_2_Cl_2_–MeOH 20:0→1:1) to yield three sub-fractions (GLC2.7.1–GLC2.7.3). The subfraction GLC2.7.1 (3.1 g) was further separated by a YMC column with MeOH−H_2_O (2:1) to obtain compound **5** (10 mg). The sub-fraction GLC2.7.3 (6.02 g) was eluted through a YMC column (MeOH−H_2_O, 2:1) to yield compound **1** (870 mg) and compound **3** (628 mg). The identities of the 7 compounds were verified by comparing their physicochemical and spectroscopic data to published values for lucidenic acid N (**1**), ganodermanontriol (**2**), lucidenic acid E2 (**3**), ganoderiol F (**4**), lucidadiol (**5**), ganodermadiol (**6**), and ergosterol (**7**) [14,37,38,39,40,41,42].

Lucidenic acid N (1): white powder; ^1^H-NMR (300 MHz, CDCl_3_), *δ_H_* 4.78 (1H, dd, *J* = 9.2, 8.4 Hz, H-7), 3.19 (1H, dd, *J* = 11.8, 4.8 Hz, H-3), 1.39 (3H, s, H-30), 1.23 (3H, s, H-28), 1.04 (3H, s, H-19), 0.99 (3H, d, *J* = 6.6 Hz, H-21), 0.96 (3H, s, H-18), 0.86 (3H, s, H-29); ^13^C-NMR *δ_C_* (35.9 (C-1), (28.3 (C-2), 78.9 (C-3), 39.7 (C-4), 50.3 (C-5), 27.9 (C-6), 67.9 (C-7), 158.8 (C-8), 144.1 (C-9), 39.9 (C-10), 200.5 (C-11), 51.5 (C-12), 46.7 (C-13), 60.5 (C-14), 218.7 (C-15), 41.8 (C-16), 47.0 (C-17), 17.6 (C-18), 18.6 (C-19), 36.4 (C-20), 18.2 (C-21), 31.8 (C-22), 31.9 (C-23), 176.1 (C-24), 28.6 (C-28), 15.6 (C-29), 24.6 (C-30).

Ganodermanontriol (2): White amorphous powder; ^1^H-NMR (300 MHz, CDCl_3_) *δ_H_* 5.52 (1H, d, *J* = 6.4 Hz, H-11), 5.42 (1H, d, *J* = 6.0 Hz, H-7), 3.86 (1H, d, *J* = 11.3 Hz, H-24), 3.50 và 3.45 (2H, d, *J* = 11.3, H-26), 1.22 (3H, s, H-30), 1.15 (3H, s, H-27), 1.13 (3H, s, H-29), 1.06 (3H, s, H-19), 0.93 (3H, d, *J* = 6.8 Hz, H-21), 0.89 (3H, s, H-28), 0.61 (3H, s, H-18); ^13^C-NMR (75 MHz, CDCl_3_) *δ_C_* 36.6 (C-1), 34.8 (C-2), 217.0 (C-3), 47.5 (C-4), 50.9 (C-5), 23.6 (C-6), 119.9 (C-7), 142.8 (C-8), 144.6 (C-9), 37.2 (C-10), 117.2 (C-11), 37.8 (C-12), 43.7 (C-13), 50.3 (C-14), 31.4 (C-15), 28.9 (C-16), 50.7 (C-17), 15.7 (C-18), 22.4 (C-19), 36.5 (C-20), 18.6 (C-21), 33.5 (C-22), 27.8 (C-23), 79.2 (C-24), 73.8 (C-25), 67.6 (C-26), 22.0 (C-27), 20.9 (C-28), 25.4 (C-29), C-30 (25.3).

Lucidenic acid E2 (3): Pale yellowish amorphous powder; [α]^25^_D_ = +836° (c = 0.45, CHCl_3_); ^1^H-NMR (500 MHz, MeOD): *δ_H_* 5.71 (1H, s, H-12), 3.32 (1H, s, COOH), 3.24 (1H, dd, *J* = 11.5, 4.5 Hz, H-3), 2.21 (3H, s, 12-OAc), 1.76 (3H, s, H-30), 1.37 (3H, s, H-19), 1.05 (3H, d, *J* = 7 Hz, H-21), 1.03 (3H, s, H-28), 0.91 (3H, s, H-29), 0.85 (3H, s, H-18); ^13^C-NMR (125 MHz, MeOD) *δ_C_* 34.4 (C-1), 28.0 (C-2), 78.1 (C-3), 41.8 (C-4), 52.7 (C-5), 37.6 (C-6), 201.2 (C-7), 153.2 (C-8), 147.2 (C-9), 40.2 (C-10), 195.6 (C-11), 80.9 (C-12), 52.7 (C-13), 59.8 (C-14), 209.0 (C-15), 38.2 (C-16), 46.5 (C-17), 12.6 (C-18), 18.2 (C-19), 33.8 (C-20), 20.6 (C-21), 31.1 (C-22), 32.6 (C-23), 177.4 (C-24), 28.7 (C-28), 16.2 (C-29), 21. 6 (C-30), 171.7 (CH_3_COO), 20.9 (CH_3_COO), (177.4 (COOH).

Ganoderiol F (4): White amorphous powder; [α]^25^_D_ = +43.0° (c = 0.13, CHCl_3_); *^1^H-NMR* (300 MHz, CDCl_3_) *δ_H_* 5.57 (1H, t, *J* = 7.6 Hz, H-24), 5.50 (1H, d, *J* = 6.2 Hz, H-7), 5.39 (1H, d, *J* = 6.1 Hz, H-11), 4.34 (2H, s, H-27), 4.22 (2H, s, H-26), 1.20 (3H, s, H-19), 1.13 (3H, s, H-28), 1.09 (3H, s, H-29), 0.92 (3H, d, *J* = 6.6 Hz, H-21), 0.87 (3H, s, H-30), 0.59 (3H, s, H-18); *^13^C-NMR δ_C_* 36.0 (C-1), 34.8 (C-2), 216.9 (C-3), 47.5 (C-4), 50.7 (C-5), 23.6 (C-6), 119.9 (C-7), 142.8 (C-8), 144.5 (C-9), 37.2 (C-10), 117.2 (C-11), 37.7 (C-12), 43.7 (C-13), 50.3 (C-14), 27.9 (C-15), 31.4 (C-16), 50.8 (C-17), 15.7 (C-18), 22.4 (C-19), C-20 (36.1), 18.3 (C-21), 36.6 (C-22), 24.4 (C-23), 131.8 (C-24), 136.7 (C-25), 67.8 (C-26), 60.2 (C-27), 25.4 (C-28), 22.0 (C-29), 25.4 (C-30).

Lucidadiol (5): White amorphous powder; [α]^25^_D_ = +86.0° (c = 1.1, CHCl_3_). *^1^H-NMR* (300 MHz, CDCl_3_) *δ_H_* 5.40 (1H, t, *J* = 6.6 Hz, H-24), 4.00 (2H, s, H-26), 3.28 (1H, dd, *J* = 11.1, 4.5 Hz, H-3), 1.67 (3H, s, H-27), 1.17 (3H, s, H-19), 1.00 (3H, s, H-29), 0.94 (3H, d, *J* = 6.3 Hz, H-21), 0.92 (3H, s, H-30), 0.89 (3H, s, H-28), 0.66 (3H, s, H-18); *^13^C-NMR δ_C_* 35.0 (C-1), 27.6 (C-2), 78.1 (C-3), 39.1 (C-4), 50.1 (C-5), 36.8 (C-6), 199.2 (C-7), 139.2 (C-8), 165.0 (C-9), 40.0 (C-10), 23.9 (C-11), 30.4 (C-12), 45.2 (C-13), 48.0 (C-14), 32.2 (C-15), 28.9 (C-16), 49.2 (C-17), 16.0 (C-18), 18.9 (C-19), 36.4 (C-20), 18.9 (C-21), 36.1 (C-22), 24.7 (C-23), 127.1 (C-24), 134.6 (C-25), 69.3 (C-26), 13.8 (C-27), 25.2 (C-28), 15.5 (C-29), 27.6 (C-30).

Ganodermadiol (6): White amorphous powder; [α]^25^_D_ = +53.0° (c = 1.0, CHCl_3_); *^1^H-NMR* (500 MHz, CDCl_3_) *δ_H_* 5.47 (1H, d, *J* = 5.5 Hz, H-24) 5.40 (1H, t, *J* = 7.0 Hz, H-7), 5.34 (1H, dd, *J* = 12.5, 6.0 Hz, H-11), 4.00 (2H, s, H-26), 3.25 (1H, dd, *J* = 11.5, 4.5, H-3), 1.64 (3H, s, H-27), 1.01 (3H, s, H-19), 0.98 (3H, s, H-29), 0.92 (3H, d, *J* = 6.5 Hz, H-21), 0.88 (3H, s, H-28, 30), 0.56 (3H, s, H-18); ^13^C-NMR (125 MHz, MeOD) *δ_C_* 35.8 (C-1), 28.2 (C-2), 79.0 (C-3), 38.7 (C-4), 49.2 (C-5), 23.0 (C-6), 120.3 (C-7), 142.7 (C-8), 145.9 (C-9), 37.4 (C-10), 116.3 (C-11), 37.9 (C-12), 43.8 (C-13), 50.4 (C-14), 31.5 (C-15), 27.9 (C-16), 50.9 (C-17), 15.7 (C-18), 22.8 (C-19), 36.1 (C-20), 18.4 (C-21), 35.9 (C-22), 24.6 (C-23), 127.0 (C-24), 134.4 (C-25), 69.1 (C-26), 13.7 (C-27), 25.6 (C-28), 27.9 (C-29), 15.8 (C-30).

Ergosterol (7): White amorphous powder; [α]^25^_D_ = −9.8° (c = 0.19, CHCl_3_); *^1^H-NMR* (300 MHz, CDCl_3_) *δ_H_* 5.57 (1H, dd, *J* = 5.7, 2.1 Hz, H-7), 5.38 (1H, dt, *J* = 5.7, 2.7 Hz, H-6), 5.22 (1H, dd, *J* = 15.4, 6.6 Hz, H-23), 5.15 (1H, dd, *J* = 15.4, 6.9 Hz, H-22), 3.63 (1H, m, H-3), 1.02 (3H, d, *J* = 6.6 Hz, H-21), 0.95 (3H, s, H-19), 0.92 (3H, d, *J* = 6.6 Hz, H-28), 0.85 (3H, d, *J* = 6.6 Hz, H-26), 0.83 (3H, d, *J* = 6.6 Hz, H-27), 0.63 (3H, s, H-18).

#### 4.4.4. Preparation of Standard Compound Solution

The standard compound solutions (1 mg/mL) were prepared by dissolving lucidenic acid N (**1**), ganodermanontriol (**2**), lucidenic acid E2 (**3**), ganoderiol F (**4**), lucidadiol (**5**), ganodermadiol (**6**), and ergosterol (**7**) in MeOH.

### 4.5. MTT Assay

Regarding the cytotoxicity tests on the cancer cell lines, the MTT assay was employed. To this end, the cancer cells were seeded in 96-well plates, incubated for 24 h at 37 °C, and the plant extracts (at concentrations ranging from 1 to 100 µg/mL) were added to each well. The mixtures were then incubated for another 48 h, and the MTT solution was subjected to the wells. The formed formazan crystals in viable cells were dissolved in DMSO, and its UV-Vis absorbance at 550 nm was measured by a microplate reader. The percentages of cell viability were calculated based on the absorbance values, in relation to the negative control (i.e., cells exposed to the control vehicle) [36].

### 4.6. Chromatography

For the HPTLC, the sample and references were applied band-wise (track distance 8 mm, band length 6 mm) on the precoated silica gel plates. Then, the plates were desiccated in a vacuum trunk for 2 h, followed by HPTLC with the low layer of dichloromethane:methanol (9:1), for 85 mm at room temperature, in a Camag twin-trough chamber. After that, the plates were visualized with 10% H_2_SO_4_ in ethanol, under heating at 105 °C. Finally, the plate was observed under UV exposure at a wavelength of 366 nm, and the HPTLC chromatograms were recorded. The corresponding digital scanning profile was generated with the self-developed software by our research team. The method was critically validated following the ICH guideline [43].

### 4.7. Statistical Analysis

The data were analyzed using the unpaired Student’s t-test between the control and compounds. Data were compiled from three independent experiments and the values were expressed as mean ± standard deviation (SD).

## 5. Conclusions

In summary, along with other analysis methods such as HPLC or GC, HPTLC is also feasible for standardization and quality control of various Linhzhi samples or Linhzhi products, based on differences in the fingerprint profiles of triterpenes on HPTLC chromatograms. The unique fingerprint profiles were observed for cultivated Linhzhi strains originated from Korea, Japan, China, and Vietnam, wild-collected Linhzhi in Vietnam, and other related Linhzhi species, including *G. applanatum*, *G. australe*, *G. clossum*, *G. subresinosu*, and *Ganoderma sp*. These distinctive triterpene components could be readily used for the rapid differentiation of these Linhzhi and related Linhzhi species. In addition, evaluation of the cytotoxicity of these species against four cancer cell lines, including A549, MCF7, PC3, and HepG2, displayed various levels of cytotoxic effect. This research contributes to the literature on the cytotoxic activities and fingerprint analysis of triterpenes by the HPTLC technique for distinguishing *Ganoderma* species from Vietnam and other Asian countries.

## Figures and Tables

**Table 1:** Summary of the tested samples and percentage yield of the methanol extracts (*w*/*w*).

No.	Scientific Name	Origin	Yield (%)
G1	*G. lucidum*	Cultivated by Linh chi Vina Company, Vietnam, Japanese Linhzhi strain.	4.05 ± 0.21
G2	*G. lucidum*	Cultivated by Linh chi Vina Company, Vietnam, Chinese Linhzhi strain.	4.22 ± 0.15
G3	*G. lucidum*	Cultivated by Linh chi Vina Company, Vietnam, Vietnamese Linhzhi strain.	3.61 ± 0.17
G4	*G. lucidum*	Cultivated by Longevity Linhzhi Farm, Chungnam Province, Korea, Korean Linhzhi strain.	3.58 ± 0.23
G5	*G. lucidum*	Cultivated by Vietnam Academy of Agricultural Sciences.	3.98 ± 0.18
G6	*G. lucidum*	Wild collected in Quang Nam-Da Nang, Vietnam, supported by Quang Nam Department of Health 2010.	4.09 ± 0.20
G7	*G. lucidum*	Wild collected in Quang Nam-Da Nang, Vietnam (grown in the dead wood of *Erythrophleum fordii*)	4.21 ± 0.32
G8	*G. lucidum*		4.05 ± 0.46
G9	*G. lucidum*		4.15 ± 0.36
G10	*G. lucidum*		4.18 ± 0.29
G11	*G. applanatum*	Cultivated by Linh chi Vina Company, Vietnam.	3.79 ± 0.22
G12	*G. clossum*	Cultivated by Linh chi Vina Company, Vietnam.	3.65 ± 0.34
G13	*G. subresinosum*	Cultivated by Linh chi Vina Company, Vietnam.	4.15 ± 0.37
G14	*Ganoderma sp.*	Cultivated by Linh chi Vina Company, Vietnam.	4.11 ± 0.40
G15	*G. australe*	Cultivated by Linh chi Vina Company, Vietnam.	3.26 ± 0.29

Extraction yield: percentage of extract yield (*w*/*w*) was calculated as: (dry extract weight/dry starting material weight) × 100.

**Table 2:** In vitro cytotoxic activity of the methanol extracts on the five tumor cell lines measured by the MTT assay ^a^.

Samples	IC_50_ (µg/mL)			
	A549	MCF7	PC3	HepG2
G1	29.3 ± 2.3	> 50	10.0 ± 2.1	10.6 ± 1.5
G2	16.8 ± 1.9	> 50	10.6 ± 1.4	24.7 ± 2.7
G3	> 50	33.8 ± 3.4	> 50	27.6 ± 3.4
G4	18.6 ± 1.3	> 50	13.5 ± 1.9	21.2 ± 2.3
G6	9.12 ± 1.5	43.6 ± 2.9	11.6 ± 1.9	21.3 ± 1.9
G11	46.3 ± 2.0	> 50	> 50	> 50
G12	24.8 ± 3.7	> 50	23.6 ± 3.2	23.5 ± 2.2
G13	15.6 ± 3.0	18.4 ± 2.9	27.8 ± 1.9	> 50
G14	17.7 ± 2.1	18.7 ± 3.1	27.7 ± 3.1	20.2 ± 2.5
G15	> 50	30.7 ± 2.8	32.1 ± 2.3	> 50

>50: No activity. ^a^ Data were compiled from three independent experiments and values are expressed as mean ± SD.

## Data Availability

Not applicable.
